# Lens-induced hypopyon uveitis as the presenting manifestation of posterior lens nucleus dislocation following pars-plana vitrectomy: case report

**DOI:** 10.1186/s12348-021-00273-z

**Published:** 2021-11-16

**Authors:** Ksiaa Imen, Ben Hadj Tahar Meriam, Sellem Ilhem, Attia Sonia, Abroug Nesrine, Khairallah Moncef

**Affiliations:** 1 Department of Ophthalmology, Fattouma Bourguiba University Hospital, Faculty of Medicine, University of Monastir, 1st June Street, Fattouma Bourguiba University Hospital, 5000 Monastir, Tunisia; 2 Loyola Stritch School of Medicine, Chicago, IL United States

**Keywords:** Pars-plana vitrectomy, Gas tamponade, Dislocated lens, Lens-induced uveitis

## Abstract

A 57-year-old otherwise healthy male presented to our department seven days following uneventful pars-plana vitrectomy with gas tamponade for a superior bullous retinal detachment in the left eye. Ophthalmic examination revealed anterior segment inflammation with hypopyon and fibrinous exudate. Intra-ocular pressure was 28 mmHg. Posterior segment evaluation was difficult to assess due to the presence of anterior capsule opacification and gas bubble. A Toxic Anterior Segment Syndrome was suspected, and the patient was treated with topical and oral corticosteroid medication in combination with anti-glaucomatous therapy. On follow-up, anterior segment inflammation and ocular hypertension improved. On day ten post-operatively, ocular ultrasonography demonstrated lens material inferiorly with attached retina. The final diagnosis of posterior lens nucleus dislocation with lens-induced uveitis was retained. The patient underwent an uneventful second vitrectomy with aspiration of the dislocated lens nucleus and sulcus three piece-lens implantation. On last follow-up, visual acuity was 20/50 with no relapsing of ocular inflammation and the retina remained reattached.

## Introduction

Posterior nucleus dislocation with an intact anterior capsule is a rare, but sight-threatening post-operative complication. It has been predominantly described in ocular trauma or inadvertent posterior capsular tear during cataract surgery and scarcely in hypermature cataracts. Although lens injuries during pars plana vitrectomy are common, data on posterior nucleus dislocation are scarce [1, 2].

We describe an unusual case of posterior lens nucleus dislocation manifesting with lens-induced uveitis following pars-plana vitrectomy with intra-vitreal gas injection for rhegmatogenous retinal detachment.

## Case presentation

A 57-year-old otherwise healthy male presented to our department with sudden mild eye pain. Seven days earlier, he had undergone pars-plana vitrectomy for superior bullous rhegmatogenous retinal detachment caused by a superior horseshoe retinal tear. Surgical procedure consisted of three-port 23-gauge pars plana vitrectomy. The trocars were introduced 4 mm posterior to the limbus managing a scleral tunnel. Core and peripheral vitrectomy was performed uneventfully. Subretinal fluid was drained under perfluorocarbon liquid. Cryopexy was applied transsclerally on the retinal tear. Perfluorocarbon/air exchange was then performed. One superior trocar was removed and the sclerotomy was sutured with 7.0 absorbable thread followed by air-SF6 20% exchange. The gas was injected continuously from the terminal trocar and the air extrusion was controlled by the other left trocar. After suturing the remaining sclerotomies, the globe was hypotonic on finger pressure. Therefore, additional 20% SF6-gas was injected 4 mm behind the limbus in the superior-temporal quadrant using a syringe with a 30G cannula.

**Fig. 1:**
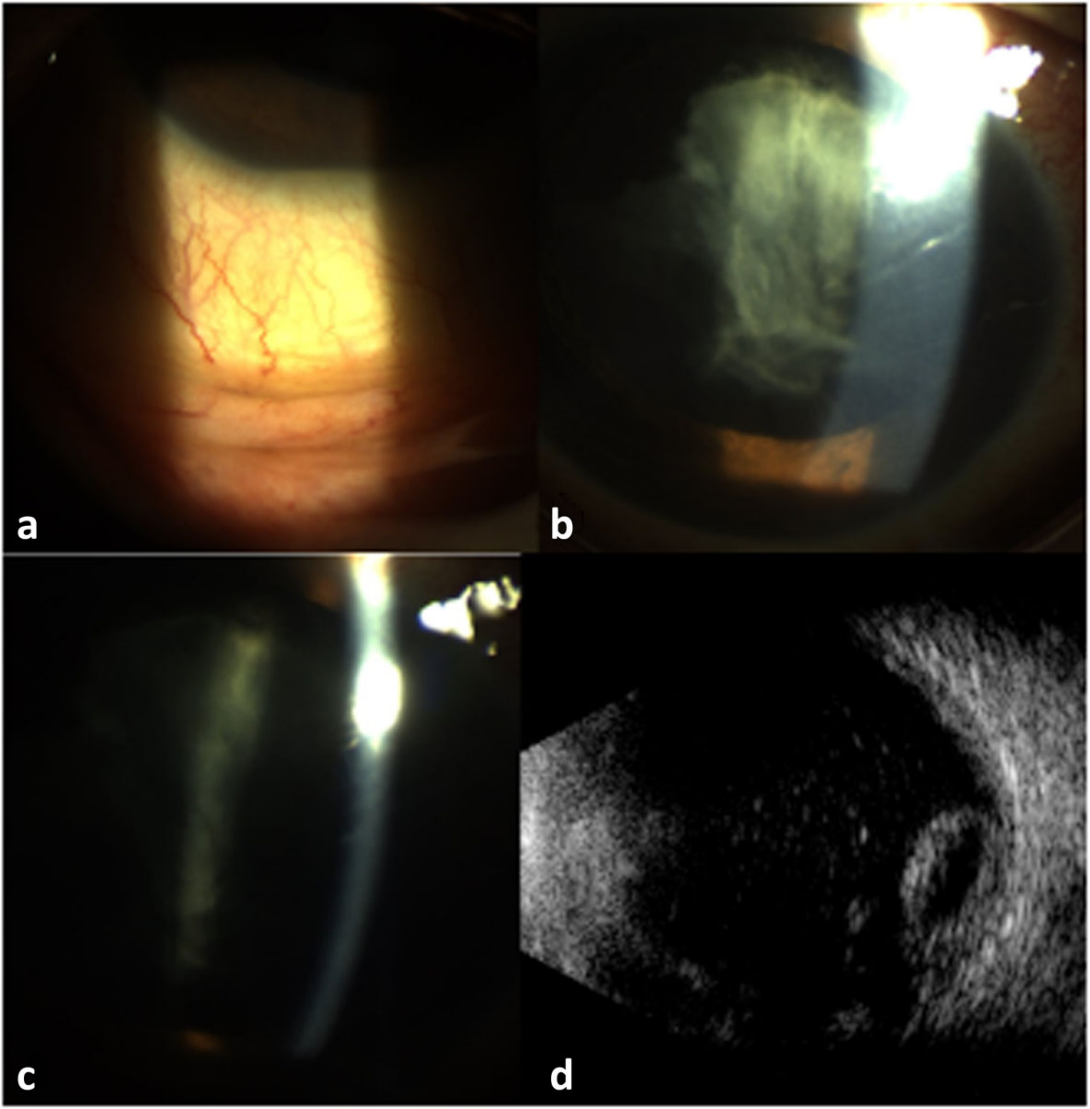
(a) Slit-lamp photography seven days after pars plana vitrectomy showing a mild conjunctival hyperemia and hypopyon. (b,c) Three days after initiation of topical steroid therapy, the hypopyon has resolved. Note the lack of typical lens capsule convexity and the residual cortical material adherent to the anterior lens capsule. (d) B-scan ultrasonography shows a globular echogenic structure in the inferior posterior vitreous cavity resting on the retina consistent with a dislocated lens nucleus

The following day, ophthalmic examination was unremarkable as the patient had positive light perception with a quiet anterior chamber, and an intra-ocular pressure (IOP) of 23 mmHg. The lens examination showed a subcapsular posterior cataract. The retina was flat posterior to the bubble. The patient was discharged on day one post-operatively and topical corticosteroid and antibiotics were prescribed. Seven days later, he presented with sudden photophobia, mild eye pain and hyperemia. On ophthalmic examination, the visual acuity was limited to positive light perception with mild corneal edema, and the IOP was 30 mmHg. A slight hypopyon was noted inferiorly with a fibrinous exudate (Fig. 1a). Posterior segment evaluation was hindered by the anterior segment inflammation. B-scan ultrasonography did not provide reliable information due to the presence of the remaining intravitreal gas bubble.

Differential diagnoses included infectious endophthalmitis and Toxic Anterior Segment Syndrome (TASS). The absence of severe pain, eyelid edema, chemosis and the mild eye redness were against the diagnosis of post-operative endophthalmitis.

The patient was treated with topical mydriatics and topical and oral corticosteroids (prednisolone, 60 mg/day). On day ten post-operatively, the anterior segment inflammation had resolved and good pupillary dilation was obtained revealing centrally clouded anterior capsule with absence of nucleus (Figs. 1 b and c). Fundus examination was obscured by opacifications behind the anterior lens capsule representing swollen lens cortex material. B-scan ultrasonography revealed a globular echogenic structure in the inferior posterior vitreous cavity resting on the retina corresponding to a dislocated lens nucleus (Fig. 1d). The diagnosis of posterior nucleus dislocation with lens-induced uveitis was then retained.

Reviewing the surgical procedure video, the posterior capsule tear had been detected when completing the gas injection with 30-gauge syringe, the globe being hypotonic and the angle of injection too anteriorly placed.

**Fig. 2:**
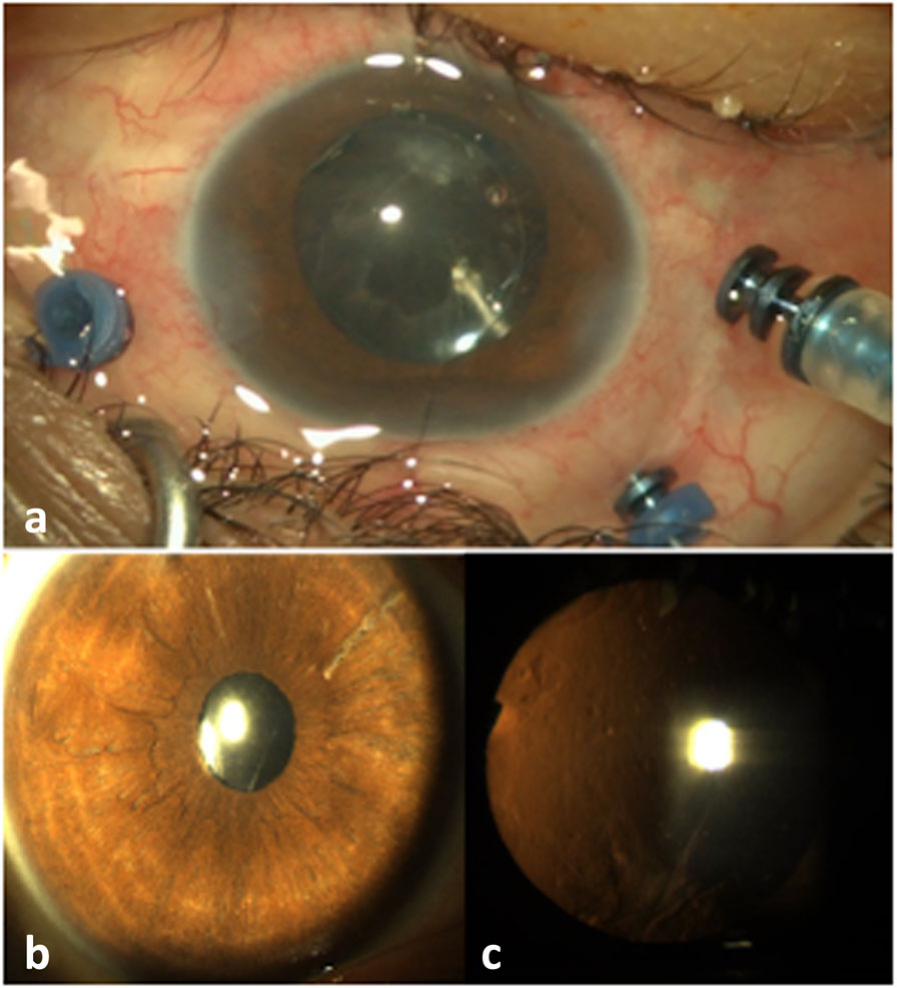
(a) Perioperative view showing the anterior capsule opacification removal from pars plana approach with the vitrector, after implantation of a sulcus-fixated 3-piece IOL. (b,c) Slit-lamp photographs one month after the second vitrectomy showing a quiet anterior segment, round pupil, and well-centered IOL

Medications were continued with a good control of ocular inflammation. Three weeks later, the patient underwent uneventful 25-gauge vitrectomy with aspiration of the soft nucleus. The anterior capsule opacification was removed by pars plana approach with the vitrector and implantation of a sulcus-fixated 3-piece intra-ocular lens (IOL) was performed (Fig. 2).

On last follow-up, visual acuity was 20/50, with a quiet eye, well-centered IOL (Fig. 2), and reattached retina.

## Discussion

This patient developed lens nucleus dislocation into the vitreous cavity following pars plana vitrectomy with gas tamponade. The lens dislocation remained quiescent during the early follow-up period until a secondary lens-induced uveitis with hypopyon became symptomatic and subsequently the nucleus dislocation was confirmed with B-scan ultrasonography.

Lens injury during intravitreal injection or pars plana vitrectomy usually leads to secondary cataract formation [1, 3–5]. Consequently cataract surgery in those cases carries a risk of intraoperative nucleus loss. However, unlike our case, spontaneous dislocation of the lens nucleus is a very rare event. Previous vitrectomy or a history of ocular trauma may have accelerated drop of the nucleus [1].

The majority of previously described patients with iatrogenic nucleus dislocation during vitrectomy had a history of ocular trauma or multiple eye surgeries to account for their lens zonulas weakness and possible posterior lens displacement, which could increase the risk for iatrogenic posterior capsule rupture [1].

Our patient presented with no history of trauma, exfoliation syndrome, or any other ocular condition associated with zonula weakening. The posterior capsule rupture in the present case probably occurred during completion of the gas injection with a 30-gauge needle due to an inappropriate angle of injection in a hypotonous vitrectomized eye that led to a posterior capsular defect and later displacement of the lens nucleus.

Unlike our case, none of the previously described patients with iatrogenic nucleus dislocation exhibited features of severe anterior chamber inflammation, such as hypopyon or fibrinous exudate. A careful clinical and ultrasonography examination was essential in our patient to rule out infectious endophthalmitis and TASS and to establish a definitive diagnosis of lens-induced uveitis with hypopyon and posterior lens nucleus dislocation.

Management in our case consisted of intensive corticosteroid and anti-glaucomatous medications followed, as previously described, by pars plana approach for the soft nucleus aspiration [1]. Consistent with previous data, implantation of a sulcus-fixated 3-piece IOL led to good visual outcome in our patient [1, 6].

Completing the gas injection at the end of pars plana vitrectomy with gas tamponade is a rather common procedure among vitreoretinal surgeons in order to obtain a complete bubble. However, the syringe should be placed cautiously and the angle should be taken into account in phakic patient to prevent lens injuries.

In any case, a diagnosis of posterior lens nucleus dislocation causing lens-induced uveitis should be considered in the differential diagnosis of post-vitrectomy uveitis with or without hypopyon.

## Data Availability

The datasets used and/or analysed during the current study are available from the corresponding author on request.

## Abbreviations

**IOL**: intra-ocular lens
**IOP**: intra-ocular pressure
**TASS**: Toxic Anterior Segment Syndrome
